# Factors influencing the suicide intervention skills of emergency medical services providers

**DOI:** 10.1080/10872981.2017.1291869

**Published:** 2017-02-24

**Authors:** Aidana Lygnugaryte-Griksiene, Darius Leskauskas, Nedas Jasinskas, Agne Masiukiene

**Affiliations:** ^a^ Psychiatric Department of Hospital of Lithuanian University of Health Sciences Kaunas Clinics, Kaunas, Lithuania; ^b^ Emergency Medical Department of Hospital of Lithuanian University of Health Sciences Kaunas Clinics, Kaunas, Lithuania; ^c^ Kaunas St. Kazimieras Lower Secondary School, Kaunas, Lithuania

**Keywords:** Training, prevention, intervention, suicides, emergency medicine, mental health, healthcare professionals

## Abstract

**Background:** Lithuania currently has the highest suicide rate in Europe and the fifth highest worldwide.

**Aims:** To identify the factors that influence the suicide intervention skills of emergency medical services (EMS) providers (doctors, nurses, paramedics).

**Method:** Two hundred and sixty-eight EMS providers participated in the research. The EMS providers were surveyed both prior to their training in suicide intervention and six months later. The questionnaire used for the survey assessed their socio-demographic characteristics, suicide intervention skills, attitudes towards suicide prevention, general mental health, strategies for coping with stress, and likelihood of burnout.

**Results:** Better suicide intervention skills were more prevalent among EMS providers with a higher level of education, heavier workload, more positive attitudes towards suicide prevention, better methods of coping with stress, and those of a younger age. Six months after the non-continuous training in suicide intervention, the providers’ ability to assess suicide risk factors had improved, although there was no change in their suicide intervention skills.

**Conclusions:** In order to improve the suicide intervention skills of EMS providers, particular attention should be paid to attitudes towards suicide prevention, skills for coping with stress, and continuous training in suicide intervention.

**Abbreviations**: EMS: Emergency medical services; SIRI: Suicide intervention response inventory

## Introduction

Data from the World Health Organization (WHO) show that Lithuania has the highest suicide rate in Europe and the fifth highest in the world. Over 1000 people commit suicide each year in Lithuania, a figure twice as high as the annual rate of traffic fatalities. Suicide is particularly prevalent among adolescents, young adults, and people of working age. Since 2006, the suicide rate in Lithuania has exceeded 30 suicides per 100 000 of population. This figure is three times higher than the European Union (EU) average.

Five key areas have been identified where suicide prevention can be improved: training programs for suicide prevention; identification methods for high-risk cases; treatment of mental disorders; limiting access to certain potential means of committing suicide; raising public awareness of the issue and disseminating information through media, public health campaigns, etc. [1]. One key factor to improving suicide prevention lies in adequate training for EMS providers. EMS providers are often the only people to have any contact with suicidal people in Lithuania. In villages and small towns, in particular, only they have to provide support as the first on the scene when a suicide risk is reported. This problem is compounded because, even following a suicide attempt, many people are not taken to the emergency room and no information on their condition is stored or forwarded on to the relevant healthcare professionals. There are no mobile crisis intervention teams and very little in the way of psychological support teams, especially in the highest-risk villages and small towns. There is also a lack of knowledge and appropriate skills with which to assess suicide risk, resulting in a generally negligent approach in such situations. There is a widespread assumption in Lithuania that people who openly proclaim their wish to commit suicide are merely being emotionally manipulative and will not see their threat through. It is common practice in emergency rooms merely to treat the wounds incurred in a failed suicide attempt and to release the patient upon his/her promise not to do it again; mental health specialists or psychological assistance are not provided. It is therefore vital that EMS providers are prepared to work in the field of suicide prevention-intervention. The actions and measures they take will directly impact the kind of help the patient receives [2]. EMS providers are constantly dealing with highly stressful situations that can be traumatizing and cause them significant psychological damage [3,4]. The mental health of EMS providers is of crucial importance, namely, the ability to recognize potentially distressing situations and to identify symptoms of stress within themselves. It is important as a preventative measure that EMS providers acquire training and techniques proven to be efficacious in stress management [5].

There is no standardized long-term training on suicide prevention-intervention in Lithuania at present. There have been isolated, one-off initiatives, but no training evaluation was carried out, including recommendations for further training. Foreign institutes, schools of higher education, and other educational institutions involved in the training of healthcare professionals similarly do not offer coherent, standardized training for suicide prevention-intervention [6,7]. There is thus a sizable disparity between the training is available via existing education programs and what sort of specialized knowledge and training should be introduced to the field of suicide intervention [8]. Studies conducted in Lithuania show that a high incidence of suicides is determined, among other factors, by a more positive attitude towards suicide as an acceptable solution to problems than in other countries. Misconceptions about suicide still abound. There is little apparent difference in attitudes towards suicide between medical staff and non-professionals. It is beyond contention that the appropriate skills, knowledge, and awareness of Lithuanian healthcare providers to deal with the potentially suicidal are insufficient: almost half of all professionals wrongly interpret the real tell-tale signs of those at risk of suicide, while two thirds of them do not trust their competence and skills to provide proper assistance to a person intent on suicide. Outdated ideas and general misconceptions are especially prevalent among those medical professionals and senior specialists who feel strong scepticism towards the usefulness of psychological support systems. An improvement in the healthcare available to those at risk of suicide is hampered by professional insularity, and a lack of knowledge, and motivation. Almost a third of the respondents felt unmotivated to participate in new training, while others agreed to participate only to the extent they were required under their stated responsibilities. However, other researches show that special training may change negative attitudes. The attitudes of those who undergo training are more favourable towards suicide prevention [9]. Most research shows that in order to achieve a steady and long-term reduction in the suicide rate, non-continuous training is not enough, and there is a need for continual, repeated, and standardized suicide prevention training. More effort should be directed towards evaluating the existing methods of training healthcare providers in this field, including making further practical recommendations and improvements, allowing for the rational allocation of resources, and instituting the long-term and systematic training of healthcare professionals [10–14].

## Materials and methods

### Aims

This is a review study that aims to examine the issue of suicide prevention-intervention in Lithuania from the perspective of support providers. The aim is to identify those areas of suicide prevention-intervention which are in most need of further research. We aim, firstly, to evaluate the attitudes of EMS providers towards suicide prevention, mental health, strategies for coping with stress, and burnout syndrome. Then we attempt to determine to what extent the suicide intervention skills of EMS providers are influenced by the following factors: sociodemographic characteristics, attitudes towards suicide prevention, mental health, strategies of coping with stress, burnout syndrome, and suicide prevention-intervention training.

### Ethical approval

Approval to conduct the research was issued by the Kaunas Regional Committee on Biomedical Research Ethics under the Lithuanian Bioethics Committee, Approval No. BE-2–15. Personal consent forms and the appropriate consent from the hospitals participating in the study were obtained.

### Study location

Utena County was chosen as the study location, because this region has the highest suicide rate in Lithuania. Four hospitals were involved in the study.

### Respondents

EMS providers (doctors, nurses, paramedics) working in hospital emergency departments and as first responders for the emergency services in Utena County; they agreed to participate in Suicide Prevention-Intervention Training (Training) and complete the survey.

### Phases of the study

1. Survey of the EMS providers prior to Training.
2. Training.
3. Second survey of EMS providers at least six months after Training.

### Training

Training was financed through structural support funds from the EU. The training was carried out by the Crisis Research Centre under the Lithuanian University of Health Sciences (LUHS). The duration of the training amounted to 32 hours: eight hours per day over four days. The training encompassed a wide range of issues, such as: attitudes towards suicide, the causes of suicidal behaviour, the psychological state of a suicidal individual, suicide risk assessment, recognisable and subtle signs of suicidal behaviour, close contact with high-risk individuals, and follow-up measures. Furthermore, the training focused on the EMS providers themselves, specifically: recognising signs of stress in themselves, how stress might affect their professional reactions and behaviour, burnout syndrome, and methods for coping with stress and conflict. The training consisted of theory, role-playing, and discussions. After the training was completed, those respondents who felt the need were provided with the opportunity to talk anonymously and on a one-to-one basis with a psychologist or a psychiatrist. They were also informed of where they could seek further psychological-psychiatric support. The training was carried out by psychiatrists and psychologists trained under Jeffrey T. Mitchell, Ph.D, a Clinical Professor of Emergency, Health Services at the University of Maryland in Baltimore County, Maryland, USA, and President Emeritus of the International Critical Incident Stress Foundation (ICISF).

### Survey of respondents

The questionnaire submitted to the respondents was anonymous, in accordance with Lithuania’s bioethical standards. Both prior to the training and six months after its completion, a survey of EMS providers was carried out, using a questionnaire developed to assess the following:

1. Sociodemographic characteristics.
2. Previous experience of suicide intervention training.
3. Ability to assess suicide risk, using a questionnaire designed by the researchers according to WHO recommendations.
4. To assess the level of suicide intervention skills we used the Suicide Intervention Response Inventory (SIRI). The SIRI is designed to assess the ability of paraprofessionals (as well as professionals) to recognize and respond to suicidal statements. The SIRI was created by Neimeyer R. A. & Maclnnes, 1981, and validated by Neimeyer & Hartley in 1986. The SIRI questionnaire consists of 24 items, each with two possible responses for helpers dealing with a suicidal individual, one of which is considered facilitative to effective intervention, whereas the other is neutral or deleterious, according to crisis intervention theory. The maximum number of points is 24. Higher scores indicate better suicide intervention skills. Available from: http://dustinkmacdonald.com/suicide-intervention-response-inventory-siri/.
5. Mental health was assessed using the Mini-International Neuropsychiatric Interview (M.I.N.I.). The following modules were applied in the survey: major depressive disorder; risk of suicide; harmful alcohol consumption and alcohol dependence syndrome; generalized anxiety disorder. Available from: http://www.medical-outcomes.com/index/mini.
6. Attitudes towards suicide were assessed using the Attitudes to Suicide Prevention Scale (ASPS). The tool is widely used to assess attitudes towards suicide during suicide prevention training. The tool consists of 14 statements, each of which is assessed on a scale of 1 to 5: 1 means ‘strongly disagree’, 2 means ‘disagree’, 3 means ‘no clear opinion’, 4 means ‘agree’, and 5 means ‘strongly agree’. The total score is calculated: the higher the rating scale, the more negative the attitude of the respondent towards suicide.
7. Burnout syndrome was measured using the Maslach Burnout Inventory (MBI). The MBI survey addresses three general areas: Emotional Exhaustion, Depersonalization, and Personal Accomplishment. Available from: http://www.mindgarden.com/117-maslach-burnout-inventory.
8. Mechanisms for coping with stress were measured using the Coping Orientation for Problem Experiences (COPE). The questionnaire consists of 60 statements. There are 15 coping subscales in the questionnaire: Positive reinterpretation and growth, Mental disengagement, Focus on and venting of emotions, Use of instrumental social support, Active coping, Denial, Religious coping, Humour, Behavioural disengagement, Restraint, Use of emotional social support, Substance use, Acceptance, Suppression of competing activities, and Planning. These are grouped into the three major coping strategies: Problem–focused coping, Emotional-focused coping, and Less useful coping. Available from: http://www.psy.miami.edu/faculty/ccarver/sclCOPEF.html.

All scales were used with the consent of the authors after a double translation of the material and the adaptation of a Lithuanian version for use in the pilot study. The questionnaire was composed and the survey was conducted at the expense of the researchers. The study contained no conflicts of interest.

## Results

Statistical data analysis was performed using data collection and analysis SPSS 20.0 (Statistical Package for Social Science 20 for Windows) software package. The premise of Continuous variable normality was verified using the Shapiro-Wilk test. For variables that did not satisfy the premise of distribution normality, the Mann-Whitney U test was used instead (in two independent samples). Statistical dependence between variables was measured using Spearman’s rank correlation coefficient. For analysis of the relationship between a dependent variable and a few independent variables, linear regression analysis was used.

Three hundred and seventy-six EMS providers underwent training. During the first survey, 376 of respondent surveys were delivered: 268 of them were returned, resulting in a response rate of 71%. During the second survey, 268 of respondent surveys were delivered, of which 226 were returned, resulting in a response rate of 84%.

Two hundred and sixty eight EMS providers participated in the study, consisting of 76 physicians, 190 nurses, and two administration representatives. The respondents were made up of seven (2.6%) men and 261 (97.4%) women, their age ranging from 22 to 84 (the average age = 47.29, standard deviation = ± 9.53). Table 1 illustrates the detailed distribution of the respondents according to their sociodemographic characteristics.

**Table 1.** Sociodemographic characteristics.

Gender	n	%
Male	7	2.6
Female	261	97.4
**Age**		
22–42	77	29.2
43–63	180	68.2
64–84	7	2.7
**Education**		
Vocation	8	3.0
College	140	52.2
Higher	120	44.8
**Position**		
Doctor	76	28.4
Nurse	190	70.9
Administration	2	0.7
**Employment period**		
Up to 10 years	26	10.6
11 – 20 years	49	20.0
21–30 years	92	37.6
31–40 years	69	28.2
41 – 52 years	9	3.7
**Workload**		
10 hours per week	2	0.7
20 hours per week	5	1.9
30 hours per week	177	66.0
40 hours per week	50	18.7
50 hours per week	25	9.3
60 and > hours per week	9	3.4

The assessment of mental health using M.I.N.I. showed that 36 (13.4%) respondents met the criteria for risk of suicide, 103 (38.4%) respondents qualified for major depressive disorder (past or present), 26 (9.7%) respondents could be classified as having a current depressive disorder, 180 (67.2%) respondents exhibited signs of a generalized anxiety disorder, 10 (3.7%) respondents indulged in harmful alcohol consumption and 5 (1.9%) met the criteria for alcohol dependence syndrome. The results demonstrate a risk of suicide among younger EMS providers (≤ 47 years old) and those with higher education (p < 0.05). The majority of the respondents at risk of suicide had been in employment for less than 25 years and had a workload of less than 30 hours per week (p < 0.05). The survey results also reveal frequent depression among EMS providers in employment for less than 25 years (p < 0.05). EMS providers who met the criteria for risk of suicide and major depressive disorder were prone to emotional exhaustion, emotional indifference, cynicism, detachment and formality in relations (p < 0.05), as well as a less adaptive strategy of coping with stress in stressful situations (p < 0.01). Respondents with generalized anxiety disorder demonstrated significantly lower personal accomplishment, higher emotional exhaustion, and a less adaptive coping strategy (p < 0,001) compared to the respondents without generalized anxiety disorder (p < 0.05).

Our analysis of burnout syndrome, factoring in the sociodemographic characteristics of the respondents, found that the younger EMS providers who had been in employment for a shorter period, demonstrate higher emotional exhaustion compared to the older EMS providers with a longer period of being in employment (see Table 2). Our assessment of suicidal ideation among EMS providers shows that higher emotional exhaustion is specific to EMS providers who have dealt with cases of suicide in their clinical work (p < 0.05). Significantly higher depersonalization was observed in those respondents who had dealt with cases of suicide in their clinical work or personal lives or who had themselves considered suicide (p < 0.01).

**Table 2.** Maslach Burnout Inventory Emotional Exhaustion scale averages of different age groups and employment periods.

Sociodemographic characteristics		n	SIRI Mean ± SD	Mean Rank	Mann-Whitney U	Z	P
Age	≤47	137	16.75 ± 8,42	141.64	7447.00	−2.02	0.04
	≥48	127	14.75 ± 8,47	122.64			
Employment period	≤25 years	123	16.91 ± 8.51	132.44	6341.50	−2.10	0.04
	≥26 years	122	14.65 ± 8.51	113.48			

Significance level (p < 0.05).

The results in Table 3 show that younger respondents with a shorter period of employment, higher level of education, and a heavier workload are significantly more effective at suicide intervention than older respondents with a longer period of employment, lower level of education, and lower workload. Statistically significant results demonstrate better suicide intervention skills among EMS providers who met the criteria for risk of suicide compared with those EMS providers who had did not meet the criteria for risk of suicide (p < 0.05). The suicide intervention skills of EMS providers with a higher expression of problem-focused coping with stress were shown to be significantly better compared to EMS providers showing a lower expression of problem-focused coping with stress (p < 0.05). We found that the suicide intervention skills prior to training significantly correlated with attitudes towards suicide prevention. Respondents who had received more exposure to suicide intervention skills prior to their training held less negative attitudes towards suicide prevention (see Table 4). One of the aims of this study was to determine the extent of the connection between the attitudes of EMS providers, burnout, stress, coping techniques, and how this influences the efficacy of their suicide intervention skills. Linear regression analysis revealed a significant connection between the attitudes of EMS providers towards suicide prevention and their suicide intervention skills. The results are shown in Table 5.

**Table 3.** Comparison between suicide intervention skills and sociodemographic characteristics.

Sociodemographic characteristics		n	SIRI Mean ± SD	Mean Rank	Mann-Whitney U	Z	P
Age	≤47	137	13.90 ± 4.41	161.54	4721.00	−6.43	<0.001*
	≥48	127	9.63 ± 5.18	101.17			
Education	Vocational/college	148	10.51 ± 5.10	114.00	5846.00	−4.82	<0.001*
	Higher	120	13.43 ± 4.93	159.78			
Employment period	up to 25 years	123	14.07 ± 4.55	150.40	4132,50	−6.09	<0.001*
	from 26 years	120	9.86 ± 4.94	95.37			
Workload	≤30 hours per week	184	11.19 ± 5.21	124.51	5889,00	−3.13	<0.01**
	≥40 hours per week	84	13.32 ± 4.93	156.39			

Significance level (p < 0.001*; p < 0.01**).

**Table 4.** Correlations between attitudes towards suicide prevention and suicide intervention skills.

Suicide intervention skills (SIRI)	Attitudes towards suicide prevention (ASPS)	
Prior to the training	Spearman correlation coefficient (r_S_)	−0.309**
After the training	Spearman correlation coefficient (r_S_)	−0.176*

Significance level (p*<0.05; p**<0.001).

ASPS: The Attitudes to Suicide Prevention Scale.

**Table 5.** Linear regression – suicide intervention skills predictive factors analysis.

Predictive factors	B	Beta	t	p
Attitudes towards suicide prevention	−0.29	−0.32	−4.8	**0.001**
Emotional exhaustion	0.07	0.11	1.60	0.11
Depersonalization	0.16	0.12	1.77	0.08
Personal accomplishment	−0.04	−0.06	−0.9	0.37
Problem-focused coping	0.04	0.07	0.84	0.40
Emotional-focused coping	−0.05	−0.07	−0.88	0.38
Less useful coping	0.08	0.07	0.97	0.33

Regression equation coefficients: correlation coefficient, R = 0.37; determination coefficient, R^2^ = 0.14;

Adjusted R square = 0.11; variance, F = 5.18; unstandardized coefficient, B; standardized regression coefficient, Beta; t, t-test.

Significance level (p < 0.001).

The dependent variable: suicide intervention skills (SIRI score) during the first survey.

In order to determine whether non-continuous training can have a significant impact on the accurate assessment of suicide risk, as well as on the better application of suicide intervention skills over the long-term, we carried out two assessments of the respondents: prior to the training and six months after completion. Our findings showed that EMS providers’ ability to accurately assess risk of suicide improved (p < 0.05). However, there was no change in the quality of suicide intervention skills six months after the training (p > 0.05). The respondents’ strategies for coping with stress and burnout syndrome, and their attitudes towards suicide prevention both prior to training and after, did not reveal any statistically significant changes (p > 0.05).

## Discussion

In our study, we challenge the significance of the fact that a considerable proportion of the respondents met the criteria for various mental disorders. A wide range of similar studies from other countries show that this phenomenon is certainly not unique to Lithuania. A systematic review and meta-analysis of 54 studies involving 17 560 physicians estimated that the prevalence of depression or depressive symptoms among physicians is 28.8%, ranging from 20.9% to 43.2%, depending on the tool used, and this increased with time [15]. A study carried out in Turkey showed that 47% of nursing staff suffered from anxiety symptoms [16], and 58.3% of nurses in critical care units in Albaha hospitals suffered from mild anxiety [17]. The prevalence of anxiety disorders among medical students and residents was found to be 72.26% [18] and 57% [19], and 41.9% [20] in other studies. The rate of suicidal ideation among medical students and young physicians was 7.5% [21], 11.2% [22,23], and 14% [24]. Out of 18% of Canadian physicians who were identified as depressed, only 25% considered getting help and only 2% actually did (Canadian Medical Association). Another study in Europe found that 78.3% of distressed hospital physicians had never sought professional help for depression/burnout [25]. Research further suggests that when faced with medical illness and psychological distress, most physicians use self-prescribed medication and other forms of self-treatment [25–29]. It appears that doctors routinely cope with stress or psychological problems through denial and avoidance, although the efficacy of this is questionable [30–33]. One possible reason for this is the perceived stigma related to openly admitting to a need for help. Doctors may fear that it could be construed as an indication of weakness or an inability to cope with professional pressures [34–36]. It is worth noting that this fear of stigmatization may develop as early as their time at medical school [37,38]. This is the first study of the psychological health of healthcare professionals to be carried out in Lithuania. Additional research is needed to determine what other factors affect the well-being of doctors, to provide insights as to how to reduce stress and burnout among doctors, to discover how best to improve the detection of behavioural health problems in all healthcare professionals, and how to facilitate their access to psychological and psychiatric support.

Our study showed that nearly half of the respondents suffered from emotional exhaustion, and this was more of a characteristic of younger EMS providers with a shorter period of employment. There is a higher risk of burnout for younger employees (19–25 years of age) or middle-aged professionals (40–50 years of age). Younger people experience burnout due to the disparity between their expectations of work and the reality of it, and through emotional strain at work [38,39].

Our results have demonstrated that problem-focused coping was associated with a decreased risk of burnout, while emotion-oriented coping was associated with an increased risk of burnout. It appears that specific coping styles are associated with a varied risk of burnout among emergency room staff. Coping-style intervention may reduce burnout, leading to improvement in both personal well-being and patient outcomes [40].

Our study shows that improved suicide intervention skills were related to age (younger people), mental health, attitudes towards suicide prevention and problem-focused coping. The results were statistically significant, and prove that EMS providers who met the criteria for being at risk of suicide possessed better suicide intervention skills compared with those who were not at risk of suicide. With regard to individual suicidal tendencies, negative as well as positive relations were found. Suicidality may have a negative impact on the professionalism of EMS providers in the form of personal problems, emotional instability, or permissive attitudes, or, conversely, it may help them in their work since having similar experiences enables better understanding and empathy [41,42]. To summarize, we can draw the hypothetical conclusion that suicide intervention skills correlate positively with attitudes towards suicide prevention: the more negative the attitude towards suicide prevention, the lower the suicide intervention skills. Studies carried out in other countries have shown a clear correlation between the lack of relevant knowledge, training deficiency, negative attitudes towards suicide, and inadequate healthcare in the field of suicide [10,43,44]. Negative attitudes affect patients and contribute to their feelings of worthlessness, despair, and rejection [45]. Most nurses and doctors working in emergency rooms are sceptical about suicide prevention [46], while ambulance personnel stated that they were not trained to work with suicidal patients [47]. Our research shows that many healthcare professionals are not able to identify those at risk of suicide or properly deal with suicidal people. This is mainly because of the following factors: a lack of relevant knowledge about suicide; negative attitudes towards suicide; a lack of skills in assessing the risk of suicide; a lack of human resources and time; a lack of support and supervision; no clear guidelines for medical institutions for recognising the risk of suicide or providing an appropriate response to the threat of it [48,49]. A study in Lithuania in 2005 assessed the readiness of various people working in education, law enforcement, and healthcare institutions to participate in suicide prevention. It found that their knowledge of suicide prevention was insufficient: almost half of those tested failed to correctly identify the characteristics typical of suicide risk; nearly two-thirds did not trust their ability to assist a person with suicidal intent. This lack of knowledge is especially prevalent among medical professionals and senior specialists who are more sceptical about psychological support as a viable form of treatment. Suicide prevention among people working in education, law enforcement, and healthcare is ineffective because of the widespread negative attitude towards suicide and the lack of motivation to improve their understanding of suicide: almost a third of respondents were unmotivated to undergo training in suicide prevention, while others agreed to participate only to the extent they were professionally bound to under the terms of their contracts [9].

Our study showed that younger respondents revealed higher rates of burnout symptoms, but their suicide intervention skills were better, while older respondents revealed lower rates of burnout symptoms, but their suicide intervention skills were worse. This discovery raises the difficult questions of professional incompetence and culturally ingrained attitudes. Because Lithuania is a post-Soviet country, there are significant differences between the values and attitudes of the older and younger generations. Older healthcare providers grew up in a different climate and received different training, especially in the field of suicide. Younger healthcare providers are more open to innovation, more receptive to new ideas, and more broad-minded. We conclude that younger EMS providers are more empathetic towards suicide and have better intervention skills, but they are also more prone to burnout. The expectations of young people are often incompatible with reality. The older generation, however, is psychologically more adept at protecting itself with healthcare workers often dissociating themselves from their experiences.

Six months after the non-continuous training, the ability to assess suicide risk factors had improved, although the suicide intervention skills of the EMS providers remained unchanged. In light of these results, we postulate that knowledge can be transformed faster than skills. Consider the data on the efficiency of suicide intervention training from other countries. In Japan a two-hour training session on suicide intervention was developed, including how to accurately assess risk factors and signs, for first-year resident physicians of all specialties. The participants’ confidence and attitudes significantly improved after the training, although the effectiveness was limited after 6 months [50]. In the United Kingdom, the STORM Project, which is a brief educational intervention for front-line health professionals in contact with suicidal patients aiming to improve the assessment and management of suicide, raised the confidence of the participants in suicide intervention [51]. However, the STORM Project may not be sufficient to reduce the population suicide rate [52]; on the other hand, a reduction in the frequency of suicidal acts was reported in Nuremberg during a two-year regional intervention [53], and a five-year regional intervention in a group of general practitioners has recently been reported to result in a greater decline in suicide rates compared with the surrounding region and national rates [10]. Our findings have been confirmed by numerous studies of suicide prevention training, namely, that non-continuous training is not sufficient in itself to achieve a steady and long-term reduction in suicide rates. Rather, there is a demonstrable need for consistent, repeated, and standardized training in suicide prevention that covers various aspects of suicidal behaviour [11–14]. We further believe that since there is no consistent, repeated, and standardized training on suicide prevention at present, Lithuania will not achieve a meaningful degree of preventative efficiency. Hitherto there have only been occasional initiatives in Lithuania, none of which were properly evaluated to test for their effect on suicide prevention. The study has shown that one-time training does not improve suicide intervention skills. Other studies from abroad have assessed the changes to suicide intervention skills following a period of training and have shown that suicide intervention skills improved immediately after training. They also showed, however, that there were no discernible changes in the quality of suicide intervention skills after a further six months if the training was not repeated [50]. A further goal of this study is the establishment of a suicide training model designed according to WHO recommendations and a detailed subsequent assessment of it. We cannot say with any certainty if attitudes towards suicide provision change after training. It remains unclear whether existing, preconceived attitudes can have a significant impact on the quality of suicide intervention training. The issues raised by this study should be subjected to more detailed research, including the provision of continuous training sessions and monitoring any subsequent changes in attitudes towards suicide prevention.

## Strengths and limitations of the study

It is the first study in Lithuania to perform a comprehensive analysis of the factors influencing the quality of emergency assistance to people at risk of suicide, and the first to assess the mental health of EMS providers using a standardized methodology. Most studies on suicide prevention-intervention have been carried out from the perspective of patients, whereas we have attempted to better understand the mind-set of emergency response providers. Two parties are involved in the process of assisting a suicidal patient, which is why it is important to assess both of them and understand how they mutually influence and affect each other. There are, however, some limitations to this study. The study was carried out in only one county and covered a small sample; the assessment was performed only twice – prior to the training and six months afterwards. In order to perform a more effective assessment, respondents should be surveyed prior to the training, immediately after the training, and one, three, and six months after the training. In order to demonstrate that repeated and long-term training improves suicide intervention skills, such training and evaluation thereof should be carried out regularly and repeatedly.

## Conclusions

Younger EMS providers demonstrated an increased risk of suicide and greater emotional exhaustion, but had better suicide intervention skills. Older EMS providers were less emotionally exhausted and had a lower risk of suicide, but had worse suicide intervention skills. Better suicide intervention skills were related to being younger, having a higher level of education, a heavier workload, more positive attitudes towards suicide prevention and problem-focused coping methods for stress. Six months after completing the non-continuous training, the assessment of suicidal risk factors had improved, however, suicide intervention skills, attitudes towards suicide prevention, and strategies of coping with stress were unchanged. In order to improve the suicide intervention skills of EMS providers, particular attention should be paid to changing attitudes towards suicide prevention, improving their skills for coping with stress, and the provision of continuous training in suicide intervention.
